# Functional growth inhibition of influenza A and B viruses by liquid and powder components of leaves from the subtropical plant *Melia azedarach* L.

**DOI:** 10.1007/s00705-018-3830-x

**Published:** 2018-04-09

**Authors:** Kuniaki Nerome, Kazufumi Shimizu, Shiori Zukeran, Yasuhiro Igarashi, Kazumichi Kuroda, Shigeo Sugita, Toshikatsu Shibata, Yasuhiko Ito, Reiko Nerome

**Affiliations:** 1 The Institute of Biological Resources, 893-2, Nakayama, Nago-shi, Okinawa 905-0004 Japan; 2 Division of Microbiology, Nihon University School of Medicine, 30-1, Oyaguchi-kamicho, Itabashi-ku, Tokyo, 173-8610 Japan; 3 Biotechnology Research Center and Department of Biotechnology, Faculty of Engineering, Toyama Prefectural University, 5180 Kurokawa, Imizu-shi, Toyama 939-0398 Japan; 4 Equine Research Institute, Japan Racing Association, 1400-4, Shiba, Shimotsuke-shi, Tochigi 329-0412 Japan; 5 Division of Gastroenterology and Hepatology, Nihon University School of Medicine, 30-1, Oyaguchi-kamicho, Itabashi-ku, Tokyo, 173-8610 Japan; 6 Department of Biomedical Sciences (Graduate School), College of Life and Health Sciences, Chubu University, 1200, Matsumoto-cho, Kasugai, Aichi 487-6501 Japan

## Abstract

We evaluated the anti-influenza-virus effects of *Melia* components and discuss the utility of these components. The effects of leaf components of *Melia azedarach* L. on viruses were examined, and plaque inhibition tests were performed. The *in vivo* efficacy of *M. azedarach* L. was tested in a mouse model. Leaf components of *Melia azedarach* L. markedly inhibited the growth of various influenza viruses. In an initial screening, multiplication and haemagglutination (HA) activities of H1N1, H3N2, H5, and B influenza viruses were inactivated by the liquid extract of leaves of *M. azedarach* L. (MLE). Furthermore, plaque inhibition titres of H1N1, H3N2, and B influenza viruses treated with MLE ranged from 10^3.7^ to 10^4.2^. MLE possessed high plaque-inhibitory activity against pandemic avian H5N1, H7N9, and H9N2 vaccine candidate strains, with a plaque inhibition titre of more than 10^4.2^. Notably, the buoyant density decreased from 1.175 to 1.137 g/cm^3^, and spikeless particles appeared. We identified four anti-influenza virus substances: pheophorbide b, pheophorbide a, pyropheophorbide a, and pheophytin a. Photomorphogenesis inside the envelope may lead to removal of HA and neuraminidase spikes from viruses. Thus, MLE could efficiently remove floating influenza virus in the air space without toxicity. Consistent with this finding, intranasal administration of MLE in mice significantly decreased the occurrence of pneumonia. Additionally, leaf powder of *Melia* (MLP) inactivated influenza viruses and viruses in the intestines of chickens. MLE and MLP may have applications as novel, safe biological disinfectants for use in humans and poultry.

## Introduction

As an important respiratory disease, influenza may have the greatest impact of all known highly communicable diseases worldwide. However, in contrast to bacterial diseases, for which various antibiotics have been developed over many decades, the most effective approach for the treatment of viral communicable diseases is establishment of vaccine strategies rather than antiviral drugs. Indeed, to prevent virus infection and related complications caused by epidemic influenza viruses, e.g. H1N1, H3N2, and B influenza viruses, trivalent vaccines have been established to combat A (H1N1) pdm 09, H3N2 subtype, and B viruses [1]. Despite major vaccination efforts, antigenic variation occurring in epidemic seasons frequently reduces the efficacy of seasonal inactivated or live vaccines in Japan and the United States of America (USA) [2, 3]. For example, although 100% of children in primary and junior high schools in the southwestern region of Japan are vaccinated, large outbreaks have occurred in these schools, indicating that influenza virus variants quite different from the vaccine strains are prevalent in this area during the spring season [2]. Moreover, in the USA, reduced vaccine efficacy was confirmed during the 2015–2016 season due to the antigenic gap between the A (H1N1) pdm vaccine strain and epidemic viruses. Importantly, the major reason for the lack of efficacy during the 2016–2017 season was that the vaccine strains were not altered from those in the 2015–2016 season [3].

After the appearance of the H7N9 virus in southern China and subsequent detection in humans, rapid vaccine development was required [4–8]. Accordingly, several vaccines have been developed, such as virus-like particle (VLP) vaccines [9] and DNA-based vaccines [10]. However, despite the use of novel vaccine strategies in humans and poultry, frequent antigenic drift or shift occurs, allowing viruses to evade the effects of vaccines. Thus, it is necessary to design new strategies to overcome the limitations of currently available vaccines. For example, recently developed neuraminidase inhibitors have played an important role in alleviating the symptoms of influenza infection; however, preventive efficacy has not yet been achieved [11]. Based on this information, novel and highly effective anti-influenza drugs are urgently needed.

As an alternative preventative strategy, researchers may consider the use of novel compounds. For example, some chemical and physicochemical compounds targeting H5N1 influenza have been reported [12, 13]. Povidone-iodine-based compounds have been shown to have antiviral and therapeutic activities when administered via intravenous inoculation [14]. Additionally, disodium cromoglycate may also inactivate influenza viruses in the lungs [15]. Effective, safe disinfectants that are mildly disruptive to influenza should be used to improve quality of life. Zhao et al. [16] reported useful antiviral compounds derived from the *Melia* tree. *Melia* compounds inhibit herpes simplex virus, flaviviruses, and *Mycobacterium tuberculosis* [17–20]. Additionally, *Melia*-derived natural resources extracted with CO_2_, ethanol, or water have been widely used in folk medicine [21].

In our studies, we also found antiviral substances in subtropical plants, including *Melia azedarach* L., which is distributed in the Okinawan area of Japan. Our research showed that *Melia* extracts exhibit a variety of anti-influenza activities. Here, we evaluated the anti-influenza-virus effects of *Melia* components and discuss the utility of these components.

## Materials and methods

### Preparation of leaf powder and its liquid extract from *M*. *azedarach* L.

Leaves of *M. azedarach* L. were provided by the forestry department of Nago City, Okinawa, Japan, dried at 65 °C overnight, and powdered in a machine used to pulverise grain (MKCA6-25; Masuko Co. Ltd., Saitama, Japan). To prepare the liquid extract (MLE), samples were suspended in nine volumes of distilled water, heated at 115 °C for 35 min, and centrifuged at 2,900 xg for 30 min in a Kubota 7780 centrifuge (Kubota, Tokyo, Japan) fitted with a Kubota AG-1K rotor. The resulting supernatant was mixed with an equal volume of ethanol (Wako, Tokyo, Japan), incubated overnight at 4 °C, and centrifuged at 2,900 xg for 20 min. Ethanol was then removed by evaporation, and the dried material was resuspended in an equal volume of distilled water and filtered through a 10-kDa membrane (Pellicon 2 Mini Filters; Millipore Corporation, Germany). The resuspended sample was adjusted to contain 10.5 mg *Melia* extract/mL and was thereafter considered to have an activity of 1 U in subsequent examinations. To obtain leaf powder (MLP), dried leaves from the *Melia* tree were powdered as described above and dried at 37 °C for a few days.

### Cells and viruses

Madin-Darby canine kidney (MDCK) cells were grown in minimum essential medium containing 5–10% foetal calf serum. A/PR/8/34 (H1N1) (PR-8), A/Okinawa/248/2009 (H1N1) pdm (Okinawa), A/Moscow/1/00 (H3N2) (Moscow), B/Yamagata/16/88 (Yamagata), and A/duck/Singapore-Q/F119-3/97 (H5N3) (Singapore) strains of influenza virus were propagated in MDCK cells or 10-day-old embryonated chicken eggs. Except for the Okinawa strain, all of the above strains were obtained from the Non-Profit Organization of Biomedical Science Association (Tokyo, Japan); the Okinawa strain was kindly provided by the Okinawa Prefectural Institute of Health and Environment. In addition, the following low pathogenic avian influenza (vaccine seed) viruses were used, which were kindly provided by and imported (Import Permit No.: 29 douken 322, issued on 12 June, 2017 by Ministry of Agriculture, Forestry and Fisheries, Japan) from Dr. R. G. Webster, St. Jude Children Research Hospital, Memphis, TN, USA: RG-A/Barn Swallow/Hong Kong/1161/2010-A/PR/8/34 H5N1 [R] (6+2): (Swallow HK H5), RG-A/Anhui/2013-A/PR/8/34 H7N9 [R] (6+2): (Anhui H7), and A/Hong Kong/308/2014 -A/PR/8/34 H9N2 [R] (6+2): (HK H9).

### Plaque assay

The infectivity of the challenge influenza viruses was adjusted to 200 PFU/mL, and 100 μL of each virus was mixed with an equal volume of MLE. Mixtures were incubated at 35 °C for 30 min and spread on 30-mm culture plates containing monolayers of MDCK cells. Subsequently, cells were overlaid with 3 mL of 1% agar containing 15 μg/mL acetylated trypsin from bovine pancreas (Sigma-Aldrich, Germany). After 3 days at 35 °C in a 5% CO_2_ incubator, the agar of the overlaid media was removed, and cells were fixed at room temperature for 1 h with 1 mL of 3.7% formaldehyde in phosphate-buffered saline (PBS), rinsed with water, and stained with 1 mL of methylene blue tetrahydrate (Wako) in distilled water. Finally, plates were rinsed with water and dried at room temperature, and plaques were counted.

### Growth inhibition and HA inactivation tests

Growth inhibition was examined on the basis of the cytopathic effect (CPE). A 0.1-mL aliquot of 10-fold-diluted challenge viruses was mixed with 0.9 mL of MLE sample, and the mixtures were incubated at 37 °C for 60 min. The mixtures were then inoculated onto monolayers of MDCK cells, which were incubated at 37 °C for 30 min.

### HA test

Viruses were two-fold serially diluted in 50 μL of PBS in 96-well U-bottom plates, and 50 μL of 0.5% (v/v) chicken erythrocytes in PBS was added and mixed. After 30 min of incubation, HA titres were determined based on the last dilution showing complete hemagglutination, as described previously [13].

### Physicochemical and morphological characterisation of viruses treated with *Melia* extract

A/PR-8 virus was purified by centrifugation at 142,190 ×g for 4 h along a 10–50% (w/w) sucrose density gradient and treated with concentrated *Melia* extract for 120 min at 37 °C. Samples were then stained with 2% phosphotungstic acid and examined by electron microscopy (H500; Hitachi, Japan). For comparison, untreated virus was purified along a 10–50% (w/w) sucrose density gradient by centrifugation at 142,190×g for 4 h in an SW 50.1 rotor, and buoyant density was determined by centrifugation at 142,190×g for 4 h along the 10–50% (w/w) sucrose density gradient. Morphological observation of the virus particles was performed using a Hitachi H7000 electron microscope, as described previously [9].

### Identification of active components in *Melia* extract

Dried *Melia* leaves (1 kg) were extracted with 5 L of distilled water, filtered through a 10-kDa membrane (Pellicon 2 Mini Filter; Millipore Corporation), dried, and further extracted for 1 h with 1 L of 1:1 CHCl_3_-MeOH (Wako). For chemical analysis, approximately 200 L of this preparation was dried and resuspended. The aqueous layer, which exhibited activity, was collected and dried, yielding 1.46 g of dark solid. The extract was fractionated by silica gel column chromatography (CHCl_3_/MeOH = 1:0, 20:1, 10:1, 4:1, 2:1, 1:1, or 0:1, v/v), and antiviral activity against PR-8 influenza virus was detected in fractions CM4:1 (730 mg) and CM2:1 (168 mg). CM4:1 (50 mg) was fractionated by high-performance liquid chromatography (HPLC) along a gradient of MeCN-0.1% HCO_2_H (74% MeCN for 0–20 min, 74–100% for 20–25 min, and 100% for 25–36 min) to yield components A (2.6 mg), B (0.5 mg), C (1.0 mg), and D (0.2 mg). Based on nuclear magnetic resonance (AVANCE 500; Bruker Daltonics K.K., Japan), mass spectrometry (MicroTOF Focus; Bruker Daltonics K.K.), and UV spectrometry (U-3210; Hitachi), the components were identified as pheophorbide a, pheophorbide b, pyropheophorbide a, and pheophytin a, respectively.

### Safety of *Melia* extract

Samples of *Melia* extract were filtered through ultramembrane filters (Millipore Corporation) with molecular weight cut offs of 10, 30, 50, and 100 kDa and sterilised using a 200-μm filter. *In vitro* toxicity was assessed based on cytopathic morphological changes (CPEs) in MDCK cells. *In vivo* mortality was evaluated in 4-week-old ddY specific pathogen-free (SPF) mice (n = 5) purchased from Japan SLC (Shizuoka, Japan). Mice were orally administered with 0.5 mL of sample, and mortality was examined daily for 7 days. Mouse and chicken experiments were approved and performed in accordance with the Fundamental Rules for Animal Experiments and the Guidelines for Animal Experiments Performed at The Institute of Biological Resources published by the Animal Welfare and Animal Care Committee including the Animal Ethics Committee of the Institute of Biological Resources, Okinawa, Japan.

### Virus clearance from air space

Samples of PR-8 (H1N1) virus were adjusted to 2 × 10^7^ PFU/mL, and 5 mL was sprayed for 10 min into a 96-L closed chamber. Subsequently, a sample of *Melia* extract was also sprayed into the same chamber for another 10 min. Virus particles were then recovered into the MEM by aspirator and infectivity was measured by the PFU method, as described previously.

### Inactivation of virus infectivity by *Melia* powder (MLP)

We prepared three stainless steel plates (560 cm^2^), which were covered (0.015 g/cm^2^) with MLP, bamboo powder (Bam), or wood shavings (Wood) and placed into the 96-L closed chamber. Then, 5 mL of Singapore virus (5 × 10^6^ PFU/mL) was sprayed onto the plate for 30 min, using a nebulizer kept in the chamber. After infection, we collected the infected powder sample into a tube from the plate. Subsequently, 9 mL of PBS was added to 1 g of the sample and kept at room temperature for 30 min to extract the reaction fluid. The same procedure was repeated for the remaining two sample plates. Prior to plaque formation tests, the three test specimens were filtered through a 0.2-μm membrane filter.

### Virus clearance test in the intestines

Different concentrations of *Melia* powder were mixed with chicken bait at ratios ranging from 0.1 to 10%, and their effects in chickens were investigated. In this test, 10-fold-diluted A/Singapore virus was also orally inoculated, and virus titres were examined in the chicken droppings for the next 2 days.

### *In vivo* antiviral activity

Female SPF ddY mice (4 weeks of age) were purchased from Japan SLC Co. Ltd. (Shizuoka, Japan). Mice were administered *Melia* extract or PBS intranasally and infected with PR-8 virus at an infectivity of 10–20 PFU (n = 5 mice per group) using a 100 μL plastic pipette tip. Mice were sacrificed on the indicated days after infection, and pneumonia was scored on a scale of 0–5 after dissection, depending on the number of infected lobes in the lung.

## Results

### Growth inhibition of influenza viruses by MLE

First, we screened subtropical plants collected in the Okinawan area of Japan for anti-influenza activity. Our results showed that MLE inactivated the HA activity of human and avian influenza viruses tested. Moreover, as seen in Table 1, four human and three avian strains of influenza virus showed markedly plaque reduction following treatment with MLE. For example, 50% plaque inhibition titres of four human influenza virus ranged from 10^3.7^ and 10^4.2^. We also examined growth inhibition of three avian vaccine candidate strains with the use of 10-fold-diluted MLE samples. It was quite evident that 50% plaque inhibition titres of avian viruses were 10^2.6^ (Anhui H7), 10^3.6^ (HK H 9), and 10^3.7^ PFU/mL (Swallow HK H 5).

**Table 1:** Screening of the anti-influenza virus activity of MLE

		Sample treatment	
Test viruses		PBS	MLE
HA subtypes	Strains	HA titre	HA titre
	1. Human strains		
H1N1	A/PR/8/34	256	‒*
H3N2	A/Moscow/1/00	64	8
	B/Yamagata/16/88	128	8
	2. Avian strain		
H5N3	A/duck/singapore-Q/F119-3/97 (H5N3)	128	‒*

Inactivation efficacy against plaque infectivity			
HA Subtypes	Strains	50% plaque Inhibition titre/ml (log_10_)	
	1. Human strains		
H1N1	A/PR/8/34	4.0	
H1N1	A/Okinawa/248/2009	4.2	
H3N2	A/Moscow/1/00	3.8	
	B/Yamagata/16/88	3.7	
	2. Avian strains		
H5N1	RG-A/Barn Swallow/HK/1161/2010-A/PR/8/34	3.7	
H7N9	RG-A/Anhui/1/2013-A/PR/8/34	2.6	
H9N2	RG-A/HK/308/2014-A/PR/8/34	3.6	

*: HA titre less than 32

### Physicochemical and morphological analyses of growth inhibition mechanisms

**Fig. 1:**
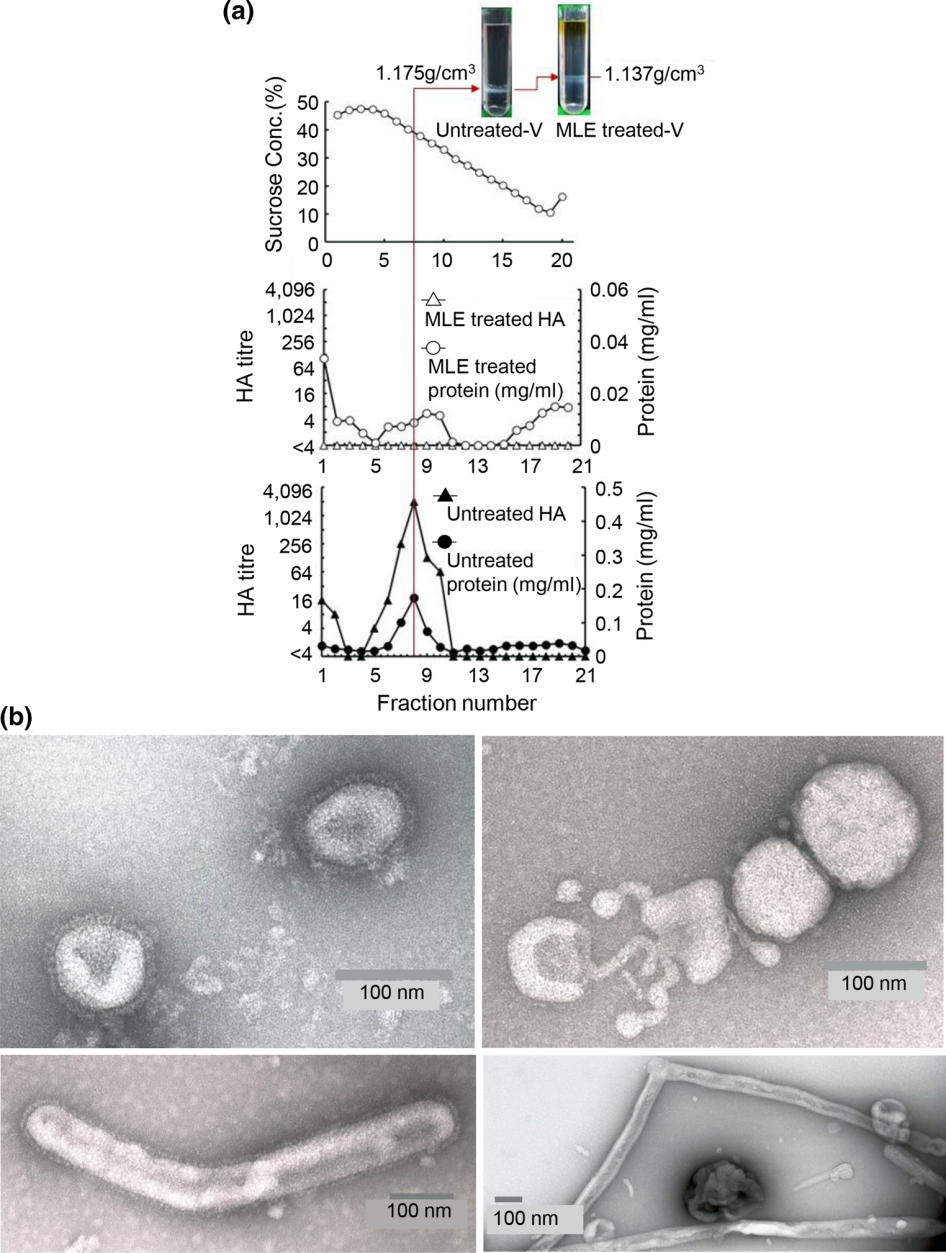
**Buoyant density analysis and electron microscopic examination of MLE-treated and untreated influenza viruses.** (**a**) Estimation of the buoyant density of A/PR-8 (H1N1) influenza virus particles treated with or without MLE. Samples were subjected to 10–50% sucrose (w/w) density gradient centrifugation at 142,190 xg for 4 h and separated into 21 fractions. Examination of the linearity of the sucrose concentration and confirmation of virus bands. Fractionation patterns of MLE-treated virus and fractionation patterns of untreated normal virus. (**b**) Electron microscopic examination of virus particles treated with and without MLE. Untreated viruses were two pictures on the left and MLE-treated viruses were two on the right

Since the HA activities of human and avian viruses were inactivated by MLE treatment, highly purified A/PR-8 virus was treated with MLE for 120 min, and the mixture was subjected to 10–50% (w/w) sucrose density gradient centrifugation at 35,000 rpm for 4 h using a SW 501 swing rotor. Notably, two different virus bands were detected in two centrifugation tubes (Fig. 1(a)). The two target virus samples were correctly analysed under a clear linear sucrose concentration, showing two different peaks (Fig. 1(a)). The first peak was detected in untreated virus specimens (Fig. 1(a)). This was different from that of the MLE-treated virus sample, in which the protein peak was present in lighter fractions 9–10; however, HA activity was not determined in these fractions (Fig. 1(a)). Moreover, the buoyant density of MLE-treated virus was decreased from 1.175 g/cm^3^ of normal virions to 1.137 g/cm^3^ (Fig. 1(a)). Conversely, protein and HA peaks of normal and MLE-treated viruses were present in fraction 7 (Fig. 1(a)). As shown in Fig. 1(b), typical normal virions of influenza virus were surrounded with dense projections (two pictures on the left). In contrast, all spikes on the surface of MLE-treated virus were completely removed from virus particles (two pictures on the right).

### Identification of chemical structures of anti-influenza components from *Melia* specimens

**Fig. 2:**
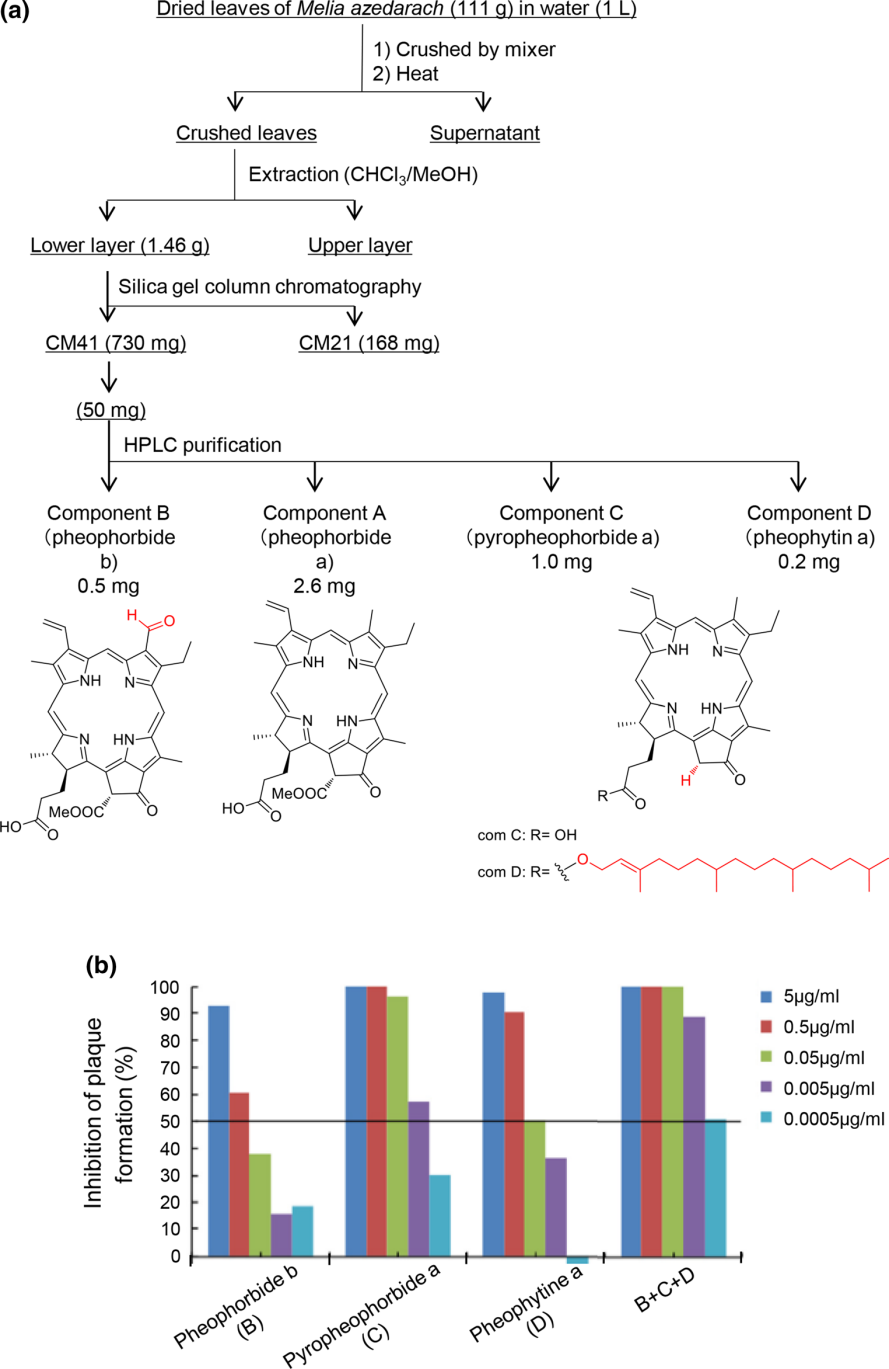
**Chemical analysis of MLE and antiviral activity of purified components.** (**a**) Identification, purification, and structure of MLE components. (**b**) Antiviral activity of each purified component, alone or in combination, against PR-8 virus

Crushed leaves were collected by centrifugation and extracted with a mixture of chloroform and methanol. The lower chloroform layer was concentrated to give a crude extract, which was further fractionated by silica gel column chromatography (Fig. 2(a)). The active fraction was then subjected to HPLC fractionation, and the antiviral activity of each fraction was evaluated (Fig. 2(b)). Structure analysis of the most active fraction revealed that the major active component was pheophorbide a, a degraded product of chlorophyll. Three more components, pheophorbide b, pyropheophorbide a, and pheophytin, were also identified as active components on the basis of mass spectrometry and UV spectral data (Fig. 2(a)). As a result, the antiviral activity of the mixture of pheophorbide b, pyropheophorbide a, and pheophytin a showed enhanced antiviral activity (Fig. 2(b)).

### Virus clearance efficacy of MLE in the closed air space

**Fig. 3:**
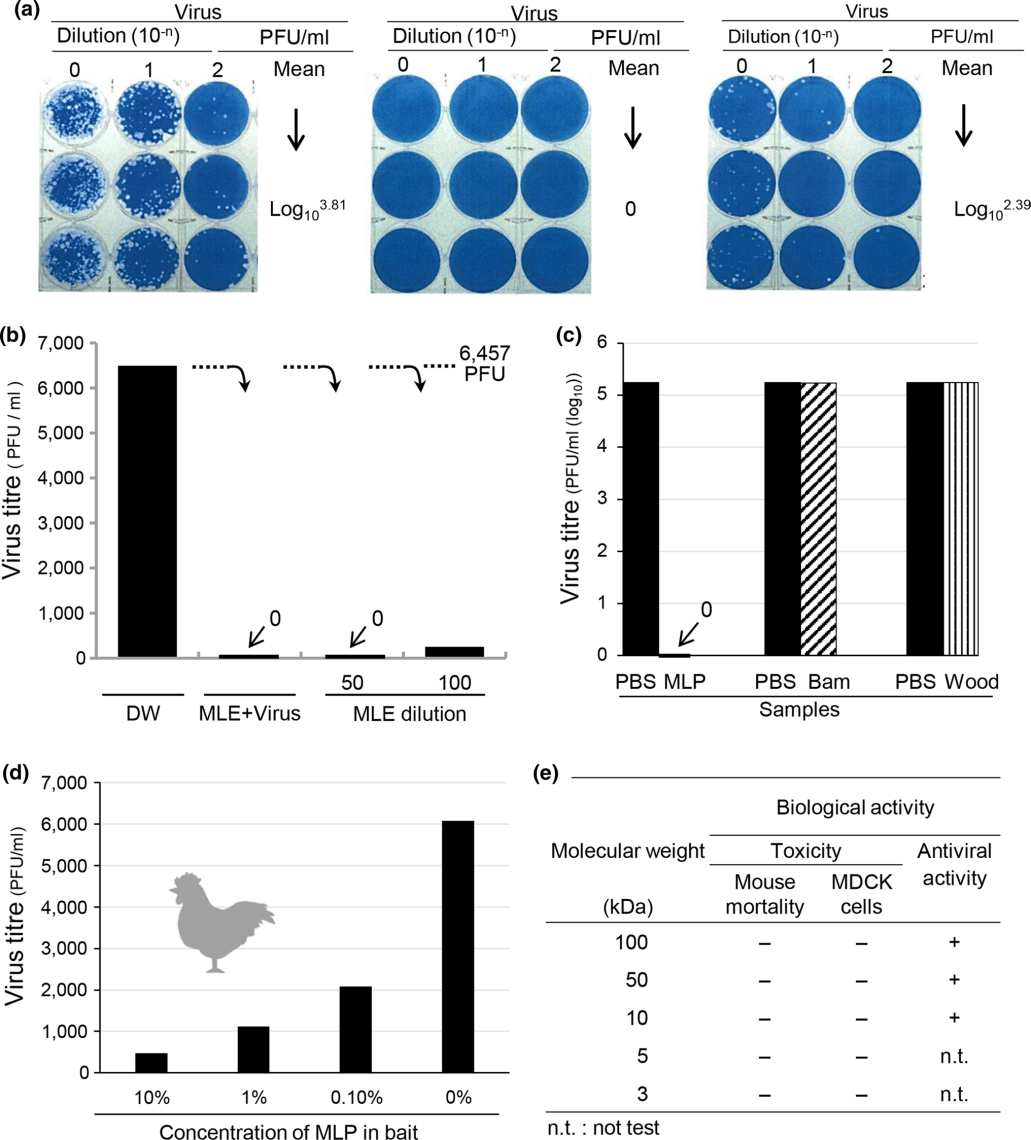
**Spraying and powder treatment efficacy of MLE and MLP against virus inactivation.** (**a**) Plaque titration of the three sprayed samples. Distilled water-sprayed virus (left), 50-fold-diluted MLE-sprayed samples (centre) and 100-fold-diluted MLE-sprayed sample (right). (**b**) Virus inactivation efficacy in the air space with MLE spray. (**c**) Virus inactivation efficacy of MLE powder (MLP), Bamboo and Wood powder. Challenge viruses possessing 5 × 10^6^ PFU infectivity were mixed with MLP on the plates and allowed to stand at room temperature. After 30 min their infectivity were measured. (**d**) Virus infectivity in the chicken intestine, which ate MLP including bait. (**e**) Safety test MLE toxicity in MDCK cells and mice

Next, we investigated virus clearance or removal efficacy in a closed air space with MLE. In this experiment, viruses (2 × 10^7^ PFU) were sprayed for 10 min in a revelation chamber in a safety cabinet (90 L air space), and 10 min later, distilled water (control) or MLE test samples were subsequently sprayed for 10 min. 10^3.81^ PFU/mL of test virus was observed in the distilled water control (Fig. 3(a), left). In contrast, the above active viruses were completely inactivated or removed by administration of 50-fold-diluted MLE (Fig. 3(a), center), whereas 10^2.39^ PFU/mL of virus remained in the air space in samples treated with 100-fold-diluted MLE (Fig. 3(a), right). These results were summarized in Fig. 3(b) together with the complete disappearance of MLE + virus sample. The finding shown in Fig. 3(a)(b), indicates *Melia* samples have the potential for virus clearance in a closed air space.

### Efficacy of MLP in influenza virus inactivation

To evaluate the virus inactivation efficacy of MLP, Singapore virus sprayed onto bamboo powder (Bam), wood savings (Wood), or MLP on the stainless steel plates was used to simulate the ground at poultry farms. The Bam and Wood test specimens showed high titres similar to virus control (PBS) titre of 10^5.2^ PFU/mL (Fig. 3(c)). In contrast, the MLP test specimen showed complete inactivation (Fig. 3(c)). Furthermore, in a chicken model infected with the low pathogenic Singapore virus, high virus titres of approximately 6,000 (10^3.78^) PFU/mL were observed in droppings, and virus titres gradually decreased as the concentration of MLP was increased from 0.1% to 10% in the bait (Fig. 3(d)). These experiments demonstrate that MLP markedly reduced virus titres in the intestine of chickens.

### Safety analysis of the MLE sample

To apply MLE in the treatment of human disease, a minimum safety requirement must be met. As shown in Fig. 3(d), partially purified MLE did not show any CPE in MDCK cells or mortality in mice.

### Prevention of experimental pneumonia in mice following MLE treatment

**Fig. 4:**
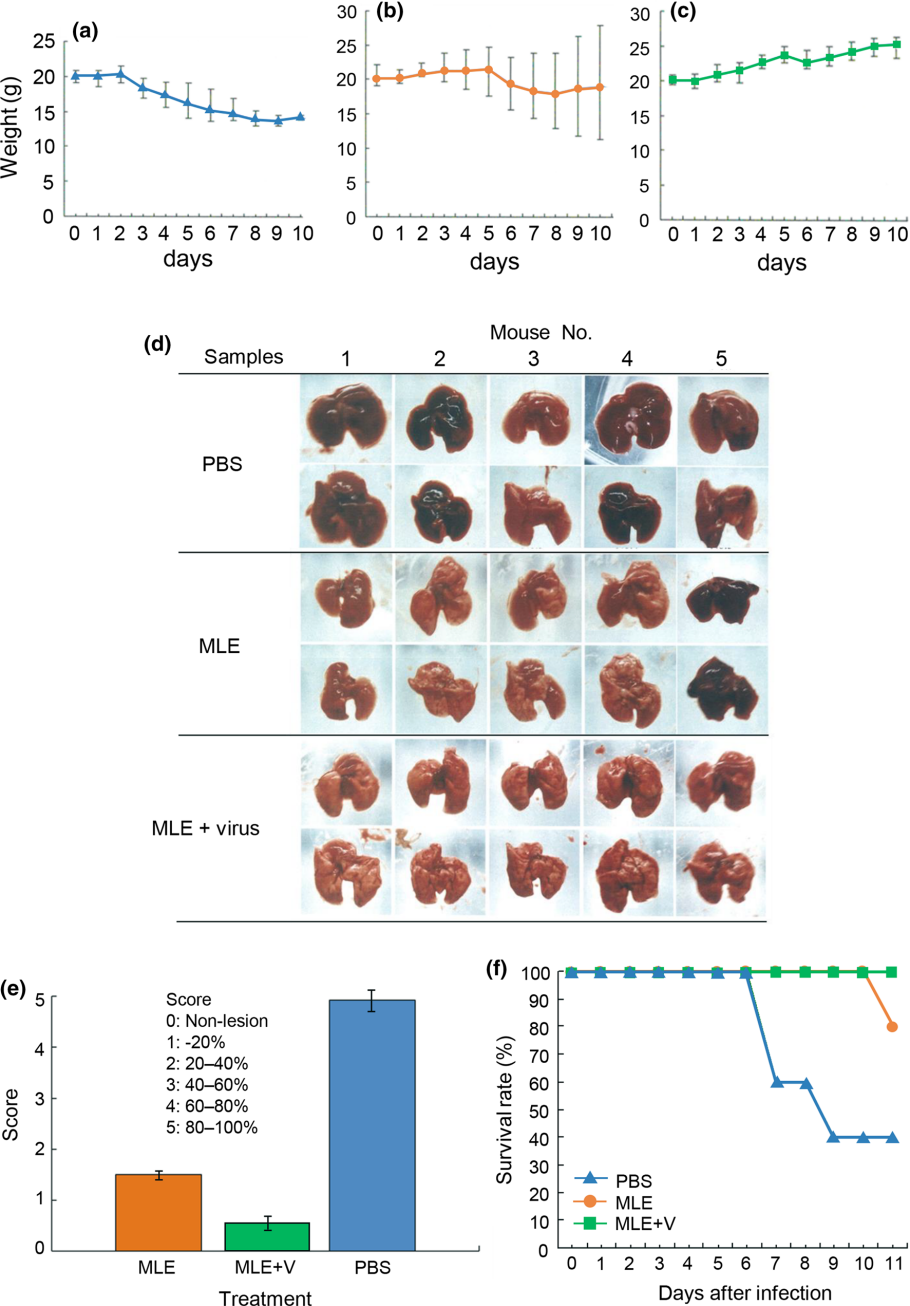
**Preventive efficacy of MLE against influenza-related pneumonia in mice.** (**a**)–(**c**) Mean body weights in mice pretreated with PBS (**a**) or MLE (**b**) or prior to infection, mice treated with MLE containing PR-8 virus (MLE + virus) (**c**). (**d**) Macropathological comparison of pneumonia occurrence in mice treated with or without MLE. Morphological and colour changes in the dorsal and abdominal sides of the lung. (**e**) Mean pneumonia score in the lungs of three mouse groups treated with MLE, MLE-virus mixture (MLE + V), and PBS. (**f**) Survival rates in the three mouse groups treated with PBS (control), MLE, and MLE + V samples

Finally, we examined the efficacy of MLE in the prevention of mouse pneumonia. As shown in Fig. 4(a), body weights of mice treated with PBS decreased from 3 days after infection, whereas mice treated with MLE (Fig. 4(b)) retained their body weight until 5 days after infection. Moreover, mice treated with a virus-MLE mixture showed a constant increase in body weight (Fig. 4(c)). Consistent with these findings, the occurrence of pneumonia in these three mouse groups differed (Fig. 4(d)). For example, four of the mouse lungs obtained from MLE-pretreated mice showed pink shading (Fig. 4(d)). In contrast, most lungs obtained from PBS control mice showed a strong black colour, indicating severe pneumonia (Fig. 4(d)). This conclusive evidence was further evaluated based on the degree of pneumonia in mouse lungs (Fig. 4(e)). Scores for pneumonia occurrence in MLE-treated, MLE+virus-treated, and PBS-treated mice were 1.5, 0.5, and 5, respectively. Consistent with the above results, MLE-treated mice showed higher preventive efficacy (80% survival; Fig. 4(f)), which was apparently different from that of PBS-treated mice (40% survival) in the absence of the MLE component (Fig. 4(f)). In contrast, the survival rate in mice treated with the MLE-virus mixture was 100%. These experiments suggest that the MLE and MLP components could prevent or alleviate the damage attributable to influenza virus infection.

## Discussion

In this study, we found that the growth of influenza A and B viruses was markedly influenced by the presence of MLE and/or MLP. Additionally, the effects of MLE and MLP on growth inhibition could overcome the risk of antigenic differences in types and subtypes of all orthomyxovirus family members. Based on these findings, MLE may be used to supplement inactivated or live influenza vaccines, which are greatly influenced by the frequent occurrence of antigenic drift or limited antigenic shift.

Influenza viruses spread from human to human through direct droplets within a short distance of 1–2 m. However, the presence of other important transmission routes should be considered. For example, transmission may occur through coughing and sneezing, during which many virus particles are scattered in the air space and human environment; the transmission of these aerosols may play important roles in the rapid spread of influenza [22].

In 1997, a commercial jet airplane experienced engine trouble and was unable to take off for approximately 4 h. A woman infected with influenza virus had boarded the airplane and suffered from a violent acute onset of influenza infection, resulting in frequent coughing [23]. In this case, Moser et al. investigated the infection rate in all passengers and the number of hours they stayed on the airplane in detail. They showed that the infection rate for 5 passengers who stayed for less than 1 hour, 9 passengers who stayed for 1–3 hours, and 29 passengers who stayed for more than 3 hours were 54%, 56%, and 86%, respectively [23], suggesting that most passengers were infected through an aerosol route originating from the single patient [23]. Thus, many influenza cases, such as those occurring in families, schools, companies, or the general social population, may be attributable to aerosol infection, although droplet infection is also an important transmission route.

When considering the above complicated transmission of aerosol influenza viruses in the living air space, countermeasures against influenza, including removal or killing of floating viruses outside the body, are essential. As described above, MLE samples could efficiently remove or kill floating viruses in a closed air space, and through this mode of virus inactivation, infection could be prevented in some individuals. Thus, these results may be important for decreasing the spread of the virus through social populations.

Based on our findings, we suggest that MLE may have applications in viral prevention. Indeed, Zhao et al. reviewed a large number of dimonoids isolated from *Melia* trees and showed that some compounds had antiviral effects on herpes simplex virus-1 and vesicular stomatitis virus [16].

In summary, our findings show that MLE could obviously remove or kill the floating virus in the air space, leading to decreased influenza infection. Moreover, MLP could inactivate or remove influenza viruses distributed on the ground at poultry farms or in the intestines of chickens, suggesting reduced rates of avian influenza virus infection at poultry farms or even in nature. Thus, *Melia*-derived compounds may be established as strategic countermeasures combinable with influenza vaccines and antiviral drugs. Vaccines and anti-influenza drugs are important measures against viral infection. In contrast, measures for the treatment of influenza virus using biological resources such as *Melia* may act outside the living body or cells, suggesting another effective strategy for treating humans and animals. Further studies are needed to assess whether such an approach may be efficacious when combined with appropriate vaccines.
